# RELATED FACTORS OF MENTAL HEALTH WELL-BEING IN THE PARTICIPANTS OF SENIOR CITIZENS' COLLEGE

**DOI:** 10.1093/geroni/igz038.1135

**Published:** 2019-11-08

**Authors:** Hayato Uchida, Shinro Matsuura

**Affiliations:** 1 University of Hyogo, Himeji, Hyogo, Japan; 2 Matsuura Clinic, Himeji, Hyogo, Japan

## Abstract

The aim of this study was to clarify the mental health well-being and its related factors of participants in senior citizen’s college. The participants were 364 persons (age 69.4+/-6.1) aged sixty years or over living in Hyogo Prefecture, Japan. We conducted a cross- sectional study that included age, family structure, employed status, self-related health, presence of chronic disorders, Instrumental ADL (TMIG index of competence score), dietary variety score (1-10), cognitive social capital, structural social capital, the scales of grandchild-grandparent relationships, Japanese version of the abbreviated Lubben Social Network Scale (LSNS-6), the frequency of going outdoors, and Mental health well-being was assessed using the Japanese version of the World Health Organization Mental Health Well-being Index-five items, WHO-5. We carried out the surveys in October in 2018. The total score of WHO-5 was 19. 6+/-4.7 among all participants. From the results of multivariate logistic regression analysis, “social isolation” (OR=4.001, 95%CI=1.584-10.043) was independently associated with low mental health well-being (WHO-5). These results suggest that, to advance the well-being of the elderly, it is necessary to develop and implement the projects which promote social functions including close relationship with others, neighbors or family members.

